# Accuracy of the fluorescence-aided identification technique (FIT) for detecting tooth-colored restorations utilizing different fluorescence-inducing devices: an ex vivo comparative study

**DOI:** 10.1007/s00784-021-03826-7

**Published:** 2021-02-13

**Authors:** W. Leontiev, E. Magni, C. Dettwiler, C. Meller, R. Weiger, Thomas Connert

**Affiliations:** 1 Department of Periodontology, Endodontology and Cariology, University Center for Dental Medicine, University of Basel, Basel, Switzerland; 2 Department of Restorative Dentistry, Periodontology, Endodontology and Pediatric Dentistry, School of Dental Medicine, Eberhard-Karls University, Tübingen, Germany

**Keywords:** Fluorescence-aided identification technique (FIT), Resin composite, Filling, Restoration, Diagnosis

## Abstract

**Objectives:**

The aim of the present study was to compare the accuracy of the conventional illumination method (CONV) and the fluorescence-aided identification technique (FIT) for distinguishing between composite restorations and intact teeth using different fluorescence-inducing devices commonly used for FIT.

**Materials and methods:**

Six groups of six dentists equipped with one of six different FIT systems each independently attempted to identify composite restorations and intact teeth on a full-mouth model with 22 composite restorations using CONV and, 1 h later, FIT. The entire procedure was repeated 1 week later. Sensitivity, specificity, and positive (PPV) and negative (NPV) predictive values, including 95% confidence intervals (CI), were calculated for CONV and FIT overall and for each device. The influence of examiner age, method, and device on each parameter was assessed by multivariate analysis of variance.

**Results:**

The sensitivity (84%, CI 81–86%), specificity (94%, CI 93–96%), PPV (92%, CI 90–94%), and NPV (90%, CI 88–91%) of FIT was significantly higher than that of CONV (47%, CI 44–50%; 82%, CI 79–84%; 66%, CI 62–69%, and 69%, CI 68–71%, respectively; *p*<0.001). The differences between CONV and FIT were significant for all parameters and FIT systems except VistaCam, which achieved no significant difference in specificity. Examiners younger than 40 years attained significantly higher sensitivity and negative predictive values than older examiners.

**Conclusions:**

FIT is more reliable for detecting composite restorations than the conventional illumination method.

**Clinical relevance:**

FIT can be considered an additional or alternative tool for improving the detection of composite restorations.

## Introduction

High-quality restorative resin composite materials, able to satisfy the high esthetic demands of contemporary patients, have been available for some time now [1]. Modern concepts for applying multiple layers of tooth-colored materials so as to match the natural tooth shade and translucency can mimic the natural tooth substance extremely well [2]. Well-matched tooth-colored restorations certainly have huge esthetic benefits but are often hard to identify later, leading to time-consuming diagnostic work, misdiagnoses, or operative difficulties in case it is necessary to remove these materials in case of re-treatment [3]. Even with a magnification aid, good illumination, and careful tooth drying, clinicians can have a hard time identifying tooth-colored restorations.

Misdiagnosis of a restoration may result in various drawbacks. A failure to detect a filling may lead to unidentified excess material or undetected new pathological findings beyond the margins [4]. Healthy tooth structure may be mistakenly removed or, conversely, composite remnants may be left behind during preparation as a result of difficulty distinguishing between tooth structure and composite [3]. Composite remnants diminish the quality of later adhesive restorations [3]. Overlooked fillings also lead to dental charting errors, which may result in false caries risk assessments and improper treatment [4]. They may also result in the falsification of epidemiological data and complicate the identification of human remains in forensic examinations [5, 6]. The implications are significant, especially in those areas where the examiners have limited equipment and time [7].

The fluorescence-aided identification technique (FIT) is a diagnostic tool to improve differentiation between composite resin restorative materials and sound tooth structure [4]. Fluorescence occurs when light is absorbed and emitted at almost the same time; however, the emitted light has a longer wavelength, making the illuminated object appear brighter [8]. Rare earth oxides added to the glass fillers of dental composites serve as fluorescent materials to adjust their fluorescent properties to optimally match the tooth structure [9, 10]. The fact that most modern commercial composite resin materials used in dentistry fluoresce at different optical intensities than enamel and dentin is essential in order to be able to perform the fluorescence-aided identification [8, 11]. The maximum fluorescence (i.e., mean of the maximum excitation wavelength) of composite was shown to be 398 ± 5 nm [8]. However, the intensity of fluorescence decreases with the age of composite resin materials [12–15].

FIT-based methods have been investigated in several studies and were shown to enhance the identification of composite fillings compared to the conventional method (CONV) because of better discrimination between the restoration material and the natural tooth structure [16–19]. Meller et al. showed that FIT leads to significantly higher accuracy of discriminating between composite fillings and intact teeth compared to the conventional method [4]. Their investigation of reliability and operator agreement also showed better repeatability, reproducibility, and higher intra- and inter-operator agreement when using FIT [4]. In other studies, FIT facilitated selective composite removal generally and from posterior teeth [20–22], trauma splint removal [23], and orthodontic bracket debonding [24–26]. In dental forensic studies, examiners detected a higher proportion of filled surfaces with FIT [7, 11, 19]. Thus, it can be concluded that FIT devices are reliable and time-saving noninvasive diagnostic tools [4, 22]. However, the influence of the different FIT devices on the detection of composite restorations remains unclear. This important knowledge could help experts propose recommendations for clinicians concerning the use of different FIT devices in daily practice.

Due to the considerations described above, the general aim of the present study was to investigate the accuracy and diagnostic predictive value of different fluorescence-inducing devices for the detection of composite restorations.

## Materials and methods

### Tooth models

The same tooth model as described in the study of Meller et al. [4] was used in this study. It consisted of 32 extracted human teeth mounted in their anatomical position on a mandibular and maxillary phantom arch. Human ethics for the use of extracted teeth were approved by local Research Ethics Committee (protocol number EKNZ UBE-15/111). Sixteen of the 32 teeth were restored with a total of 22 composite restorations using different commercially available resin composite materials and hand-modeled by means of the conventional layering technique. Of the 22 restorations, ten were placed in mandibular teeth and twelve in maxillary teeth. The restored surfaces were finished and polished using rubber polishers and brushes under water spray. Compared to the original model used by Meller et al. [4], an additional composite restoration was placed (due to a defect) in tooth no. 22 (Ceram.x Spectra ST-HV A2, Dentsply Sirona Deutschland GmbH, Bensheim, Germany). In the present study, the models were stored in 0.9% saline solution for a total of 5 years. All restorations were rated as sufficient regarding margin quality and surface texture.

### Spectral analysis

A spectral analysis for all six tested FIT devices was performed utilizing a spectrometer (AvaSpec 2048, Avantes BV, Apeldoorn, Netherlands) and the associated software (AvaSoft, Avantes BV, Apeldoorn, The Netherlands). The FIT devices were mounted in a distance of 5 cm to the detector collection lens. Details for different devices are given in Table 1.

**Table 1:** FIT devices and their properties regarding the optical emission

Device	Type	Spectral bandwidth (nm)	Peak wavelength (nm)	Color temperature (K)	Illuminance (lx)	Power received^a^ (μW/mm^2^)
DIObright	Head-lamp	398–414	407	1870	1720	506
D-Light Pro	Hand-lamp	400–411	406	1850	142	34
Dia-Stick	Hand-lamp	395–411	399	1747	0.47	0.5
SIROInspect	Hand-lamp	397–411	404	1893	102	56
D-Light Storz	Head-lamp	393–429	422	1872	6161	982
VistaCam	Camera-computer-system	400–413	407	1844	26	4.5

^a^In a range from 380 to 780 nm

### Examination procedure

Thirty-six examiners, all of them were general dentists, participated in the study. The gender distribution was even (18 males and 18 females). The examiners were divided into two age groups for the analysis: younger (age less than 40 years) and older (age ≥ 40 years). The ratio of younger to older examiners was 29:7. An Ishihara test was performed beforehand to exclude examiners with possible color impairment. One female participant (> 60 years) was excluded from the final analysis for being an extreme outlier in terms of her CONV and FIT results. All examiners were instructed and supervised by the same person to guarantee compliance with the study protocol.

After the models were mounted in a dental mannequin, they were rehydrated with water from a multifunctional syringe every 60 s to prevent dehydration and related changes in tooth color. The investigators were requested to identify and mark the extent of each composite restoration on a dental chart. Each examiner had a dental mirror, a curved dental explorer and a multifunctional syringe at their disposal. Conventional illumination was from the dental unit light source (LEDview, Sirona Dental Systems GmbH, Bensheim, Germany) with a brightness of 27.000 Lux and a color temperature of 5700–6900 K. In the FIT-setup, the following six different fluorescence-inducing sources have been used: DIObright Prototype (JADENT Dentalvertrieb GmbH, Aalen, Germany), D-Light Pro (GC, Lucerne, Switzerland), Dia-Stick (I.C. Lercher GmbH & Co. KG, Stockach, Germany), SIROInspect (Dentsply Sirona, York, Pennsylvania, USA), D-Light 20133220 (Karl Storz SE & Co. KG, Tuttlingen, Germany), and VistaCam iX (Dürr Dental, Bietigheim-Bissingen, Germany). These devices represent different types of systems (hand lamp, head lamp, and camera-computer-system). Besides the prototype (DIObright), they were all available on the market in Switzerland at the time the study was performed.

**Fig. 1:**
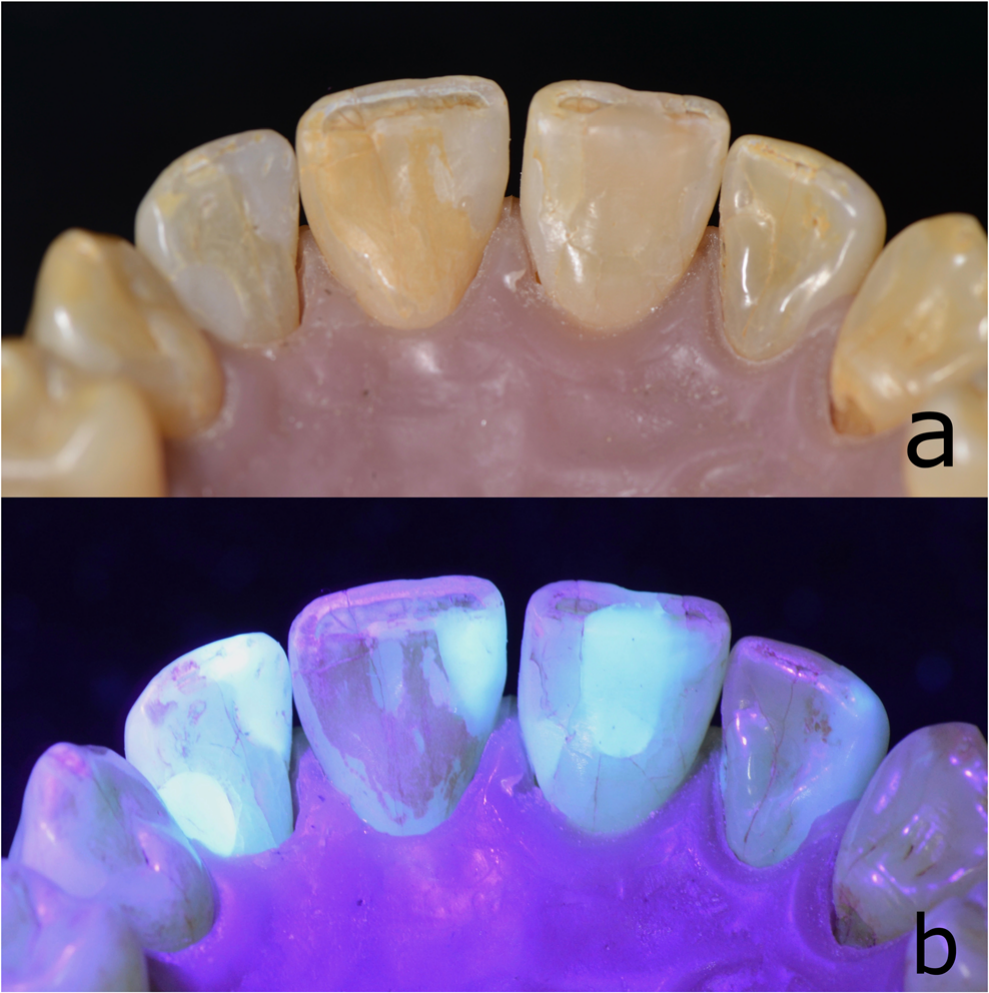
Tooth model (**a**) illuminated by conventional light source and (**b**) illuminated by FIT device

Details for the devices are presented in Table 1. All examiners wore clear safety-glasses with UV protection (uvex i-5, UVEX Arbeitsschutz AG, Basel, Switzerland). The tooth model illuminated by the conventional light source and by a FIT device is shown in Fig. 1.

All examinations for FIT and CONV were performed in the same room under the same ambient light conditions (examinations at the same daytime with no direct solar irradiation, 500–800 Lux).

Each FIT device was tested by six randomly assigned examiners, each of whom attempted to distinguish between composite restorations and intact teeth under conventional illumination and, 1 h later, under fluorescent-inducing light (λ = ~405 nm) using the devices already mentioned above. The entire procedure was repeated 1 week later, yielding a total of 144 examinations (6 groups × 6 examiners × 2 FIT × 2 CONV). Subsequently, all 144 dental charts obtained from the completed examinations were evaluated by two independent dentists who were not involved in the study design or the diagnostic identification.

### Statistical analysis

Statistical analysis of the data was performed using SPSS v.26.0 (IBM Corp., Armonk, NY, USA). Sensitivity, specificity, positive predictive value (PPV), negative predictive value (NPV), and 95% confidence interval (CI) values for the accuracy of conventional illumination and FIT overall were calculated, and the mean values were compared using the *t*-test. Based on the sensitivity and specificity values, Bayes’ theorem was used to calculate PPV and NPV dependent on prevalence (0–100%). Sensitivity, specificity, positive, and negative predictive values were also calculated for each FIT device tested. A multivariate analysis of variance (MANOVA) was utilized to assess the influence of examiner age (group A < 40 years; group B ≥ 40 years) and method (conventional method vs. FIT with a DIObright, D-Light Pro, Dia-Stick, SIROInspect, D-Light Storz, or VistaCam) on sensitivity, specificity, PPV, and NPV. This was followed by Bonferroni post hoc tests for the method. The level of significance was set at *α*=0.05.

## Results

**Fig. 2:**
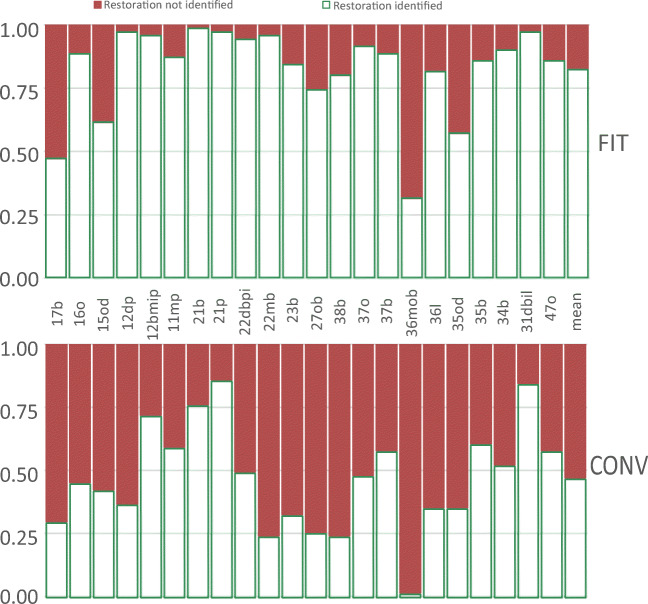
Rate of identification of restorations using CONV versus FIT

Distribution rates of identified and non-identified restorations for each restoration and method are presented in Fig. 2. Overall, the accuracy of identifying a composite restoration (sensitivity) was significantly higher with FIT (84%; CI 81–86%) compared to CONV (47%; CI 44–50%); *p*<0.001. Also, the accuracy of identifying sound tooth-structure as “non-restored” (specificity) was significantly higher with FIT (94%; CI 93–96%) than with CONV (82%; CI 79–84%); *p*<0.001.

**Fig. 3:**
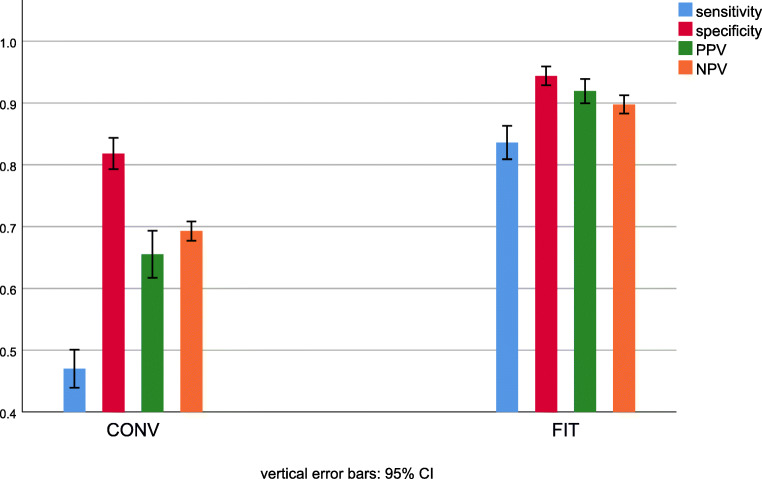
Mean values and 95% confidence intervals for sensitivity, specificity, positive predictive value (PPV) and negative predictive value (NPV) for FIT and CONV

**Fig. 4:**
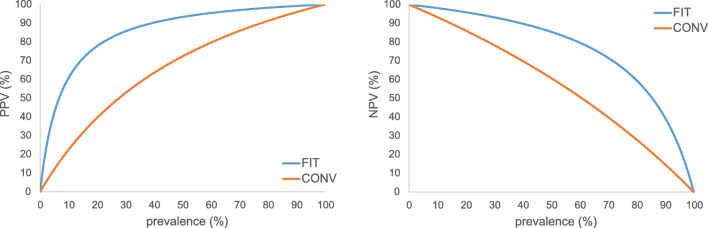
Calculated positive (PPV) and negative predictive value (NPV) for the conventional (CONV) and FIT

The probability of a site diagnosed as a filling actually being a composite restoration (positive predictive value) was significantly higher with FIT (92%; CI 90–94%) than with CONV (66%; CI 62–69%); *p* < 0.001. Conversely, the probability of a tooth diagnosed as intact actually being non-restored (negative predictive value) was higher with FIT (90%; CI 88–91%) than with CONV (69%; CI 68–71%); *p*<0.001. All four calculated values are presented in Fig. 3. Predictive values for prevalences of restored teeth that differ from the prevalence in this studies tooth model are shown in Fig. 4.

**Fig. 5:**
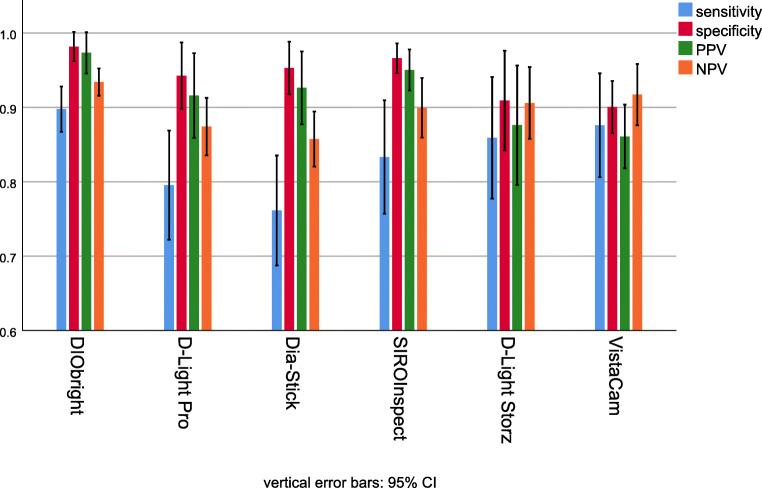
Mean values and 95% confidence intervals for sensitivity, specificity, positive (PPV) and negative predictive value (NPV) for the different FIT devices

There were differences between the different FIT lamps regarding sensitivity, specificity, PPV, and NPV. The respective mean values and 95% confidence intervals are presented in Fig. 5.

The multivariate analysis of variance showed significant differences between younger (aged < 40 years) and older examiners (aged ≥ 40 years) when considering the variables sensitivity (*p*=0.037) and negative predictive value (*p*=0.012). Younger examiners achieved higher sensitivity (mean over all methods: 66% vs. 60%) and NPV values (80% vs. 76%). The effect sizes for both variables were small: sensitivity (η_p_^2^=0.03) and negative predictive value (η_p_^2^=0.05).

All four variables were significantly affected by method (*p*<0.001), which had a large effect size (η_p_^2^=0.37). Additionally, there was significant interaction between age and method regarding sensitivity (*p*=0.036), with a medium effect size (η_p_^2^=0.09).

Bonferroni post hoc tests showed a significant difference between the conventional method and all FIT devices for sensitivity and positive predictive value (*p*<0.001). Regarding specificity, there was a significant difference between the conventional method and all FIT devices except VistaCam iX (*p*<0.05). Negative predictive values differed significantly between the conventional method and all FIT devices (*p*<0.001). Additionally, the NPV for DIObright was significantly higher than that for Dia-Stick (93% vs. 86%) (*p*=0.043).

## Discussion

Compared to the conventional method, FIT showed a significantly higher accuracy in distinguishing between composite restorations (sensitivity) and intact teeth (specificity). Also, the probability of a filling (PPV) or intact tooth (NPV) being diagnosed correctly was significantly higher with FIT.

The accuracy of a diagnostic method provides useful information if the tooth restoration status (filled or intact) is known. As the restoration status is generally unclear in daily practice, predictive values are more informative for this purpose. The predictive values in this study were calculated based on an actual filling prevalence of 42%. Since the prevalence may differ demographically, predictive plots were calculated (Fig. 4).

Fluorescent-inducing pigments are added to composites to improve the esthetic integration of tooth-colored restorations [9, 27]. The aim is to mimic the usual fluorescence properties of natural teeth to achieve the desired chameleon effect [8]. Nonetheless, changes in the UV components of daylight and artificial light can influence the color of resin composites restorations when compared to the surrounding sound natural tooth structure [28]. This can lead to a so-called fluorescence-induced illuminant metameric failure of composite materials, thus making a restored tooth more readily distinguishable from the adjacent natural tooth structure [8].

Although the observed values are more specific and significant than those of the previous trial with the same study design [4], the accuracy and predictive values for FIT are lower in the present study. The accuracy of the conventional method is higher in the present study than in the study by Meller et al. [4]. Changes related to the aging of composite restorations may explain this discrepancy. Resin composite restorations 5 years of age and older may be easier to detect by the conventional method than new restorations. It is also known that aging has a negative influence on the fluorescence properties of composite fillings [12, 15], which makes it even more difficult to detect them with the aid of fluorescence. In vitro aging cannot be compared to physiological aging. However, even if some reduction of the fluorescence signal occurs with aging, the evidence suggests that composite fillings still fluoresce more than the adjacent natural tooth structure [15].

Compared to the present study, examinations in the study by Meller et al. were performed under identical light conditions in a dark room illuminated only by artificial light. The examiners wore yellow-tinted glasses to enhance contrast. In the present study, the setting was chosen to be closer to daily practice, the examiners wore standard clear safety glasses, and examinations were performed under daylight conditions in the examination room. Therefore, daylight may have interacted with the light beams of the FIT devices and weakened their effects. Also it might be more difficult to distinguish the resin composite restorations from the tooth structure when wearing clear instead of yellow-tinted glasses, explaining the lower values for the sensitivity and PPV achieved in this study compared to Meller et al [4].

Additionally, the difference between the two studies regarding conventional method outcomes could be due to age-related differences between the groups of examiners. Whereas only post-graduate dentists participated in the present study, dental students were also allowed to participate in the previous study. Therefore, in the previous study, the average accuracy of detecting a filling or intact tooth by the conventional method may have been lower because of the lack experience of the student examiners.

Visual acuity varies highly between individuals due to various factors, including age [29]. Good vision is important not only for performing manual tasks with precision but also for achieving adequate diagnostic assessments [29]. Limitations due to natural changes in the eyesight, particularly the loss of accommodation (presbyopia), generally begin at the age of 40 years [29]. Therefore, examiners in the present study were divided into two age groups: younger and older (age ≥ 40 years). There were significant differences between the two groups. Younger dentists exhibited higher sensitivity in detecting composite fillings and a higher probability for a tooth site diagnosed as sound to actually be non-restored (negative predictive value). This is in accordance with the results of a study by Eichenberger et al., where dentists younger than 40 showed significantly higher visual acuity [29].

Accuracy and predictive values between the conventional method and all examined FIT devices differed significantly except for VistaCam iX. VistaCam iX achieved no significant difference in specificity. Additionally DIOBright showed a significantly higher probability to diagnose a non-restored tooth as sound than Dia-Stick. When comparing different FIT devices, devices with a narrower spectral bandwidth and comparably higher power output performed better, nevertheless all FIT devices achieved significantly higher values compared to CONV in nearly all categories and differences among the tested devices were little. Thus, it seems that different fluorescence-inducing light systems may be used for FIT, even though some of the devices were primarily designed for other purposes, such as caries detection. Certain systems require the use of an intraoral camera and computer monitor, which makes diagnostics not only expensive but also tedious. However, FIT does not require any expensive devices: a simple light source with a wavelength of approximately 400 nm can be used for the FIT method [8]. Certain fluorescence-inducing devices can be set up as a conventional headlight system that provides a clearly defined light spot large enough to illuminate the entire oral cavity [4]. This is probably the most straightforward way to perform fluorescence-aided identification.

Further advantages of the FIT method have been shown in previous studies. The complete removal of well-matched tooth-colored restorations can be challenging [3]. Fortunately, FIT is improving the clear recognition and detection of the margins and extent of composite fillings [4]. Notably this is relevant when removing and replacing resin composite restorations since the use of FIT provides a minimally invasive preparation with minimal overpreparation and, at the same time, low amounts of remnant restoration material [22]. In forensic science as well as in daily practice, economic aspects of the time required for diagnostics are rather important factors to consider [4]. Studies have shown that the FIT method of detecting composite restorations is almost twice as fast as the conventional method [4]. Besides, FIT requires neither a dental explorer for visual-tactile examination nor previous drying of the teeth, and FIT almost always works, even when obstructive factors such as saliva or plaque are present [4]. Meller et al. also tested the accuracy of FIT in undergraduate dental students compared to a control group of dentists. It could be shown that no working experience is needed for FIT and no prior training with FIT is necessary [4].

FIT has proven to be a diagnostic method with a high accuracy (sensitivity and specificity) for differentiating between composite fillings and intact teeth. Additional characteristics, such as repeatability, reproducibility, and inter- and intraoperative agreement, have been investigated in previous studies, which produced very satisfactory results [4]. The high repeatability values indicate that FIT produces similar results when repeated within a short period of time, and therefore, the test does not have to be repeated to attain higher accuracy [4]. The high reproducibility values show that the FIT method achieves the same accuracy when repeated under different conditions (e.g., with demographic or regional differences) or over a longer period of time [4].

The present study, like the previous one [4], showed that the conventional method of composite restoration detection yields unsatisfactory results. This suggests that existing dental records and dental epidemiological data probably contain errors due to the poor specificity and sensitivity of the conventional method. Meller et al. [4] also showed that the conventional method results in poor inter-examiner agreement. This problem is exacerbated by the fact that, nowadays, most patients prefer tooth-colored restorations, and insufficient metallic restorations are often replaced by composite resin restorations [30, 31].

Even if performed under ideal circumstances, the evidence indicates that the conventional method is not a sufficient diagnostic tool. When examiners in the present study used the conventional method, approximately every third diagnosis that a tooth was restored or non-restored was incorrect. This study proved that FIT increases the diagnostic power of composite restoration identification. Additionally, FIT is a fast, noninvasive method that requires no previous training and is not affected by other factors, such as saliva and plaque. FIT can be added to the standard diagnostic repertoire in dental practice as well as in undergraduate and post-graduate dental education.

## Conclusions

The FIT method is a fast, noninvasive approach to composite restoration identification without the need of previous training. FIT can be performed in addition to conventional illumination for improved differentiation between composite fillings and intact teeth.

Different devices emitting fluorescent light at the required wavelength of approximately 400 nm can be used for the FIT method.

Younger dentists achieve higher sensitivity and negative predictive values in distinguishing composite fillings from intact teeth than older dentists.
